# Sex-specific association between body mass index and cerebral microbleed progression in adults aged 50–85 years

**DOI:** 10.3389/fneur.2025.1624905

**Published:** 2025-09-08

**Authors:** Cindy W. Yoon, Young Ju Suh, Byeong C. Kim, Young Chul Youn, Jee Hyang Jeong, Seong Hye Choi

**Affiliations:** ^1^Department of Neurology, Inha University College of Medicine, Incheon, Republic of Korea; ^2^Department of Biomedical Sciences, Inha University College of Medicine, Incheon, Republic of Korea; ^3^Department of Neurology, Chonnam National University Medical School, Gwangju, Republic of Korea; ^4^Department of Neurology, Chung-Ang University College of Medicine, Seoul, Republic of Korea; ^5^Department of Neurology, Ewha Womans University College of Medicine, Seoul, Republic of Korea

**Keywords:** sex, cerebral microbleeds (CMB), body mass index, cerebral small vessel disease, women

## Abstract

**Background and purpose:**

Previous studies have demonstrated sex differences in the association between body mass index (BMI) and hemorrhagic stroke. Cerebral microbleed (CMB) is a clinically important marker of bleeding-prone microangiopathy, which is associated with a risk of hemorrhagic stroke. No study has evaluated sex differences in the relationship between BMI and CMB. In this longitudinal study, we aimed to conduct sex-stratified analyses to assess whether sex modifies the effect of BMI on CMB progression.

**Methods:**

The database of the CHALLENGE study (Comparison Study of Cilostazol and Aspirin on Changes in Volume of Cerebral Small Vessel Disease White Matter Changes), which enrolled patients aged 50–85 years with cerebral small vessel disease, was analyzed. Of the 256 subjects, 189 who underwent a 2-year follow-up brain MRI scan were included in the analysis. We used a generalized linear mixed model with a negative binomial distribution to assess the association between BMI and the 2-year change in CMB count, and conducted sex-stratified analyses to account for potential sex-specific effects.

**Results:**

A total of 65 men and 124 women were analyzed. In the sex-stratified negative binomial model, a significant association was observed in women but not in men. In women, each 1 kg/m^2^ increase in BMI was significantly associated with a decrease in the 2-year change in the number of total CMBs after adjustment for age and baseline CMB count [*β* = −0.120, 95% confidence interval (CI): −0.202 to −0.037, *p* = 0.005]. When CMBs were categorized into lobar and deep/infratentorial regions, significant associations were observed for both lobar (*β* = −0.114, 95% CI: −0.213 to −0.015, *p* = 0.024) and deep/infratentorial CMBs (β = −0.123, 95% CI: −0.222 to −0.023, *p* = 0.015). By contrast, no significant associations were identified between BMI and the 2-year change in CMB counts in men (all *p* > 0.05).

**Conclusion:**

Higher BMI in later life was associated with less progression of CMBs in women, but not in men.

## Introduction

Obesity is known as a risk factor for ischemic stroke in both men and women (1). However, previous studies have shown differences in hemorrhagic stroke between men and women (1, 2). In the UK Biobank cohort study, obesity was associated with an increased risk of hemorrhagic stroke only in men but not women (1). Moreover, a UK prospective study found that higher body mass index (BMI) was associated with a decreased risk of hemorrhagic stroke in women (3). In terms of the location of intracranial hemorrhage (ICH), a previous study found that obesity increased the risk of deep ICH but had no significant effect on the risk of lobar ICH (4). However, it is not well known whether there are sex differences according to the ICH location or underlying pathophysiology.

Cerebral microbleed (CMB) is a clinically important cerebral small vessel disease (cSVD) marker of bleeding-prone microangiopathy, which is associated with a risk of hemorrhagic stroke (5). The location and distribution of CMBs are considered to reflect their underlying pathology (6). Specifically, deep CMBs are presumed to be caused by cerebrovascular risk factors (e.g., hypertensive angiopathy), whereas lobar CMBs reflect cerebral amyloid angiopathy (CAA), especially in cases of strictly lobar CMB distribution (6, 7). Previous studies examining the relationship between CMB and BMI have reported inconsistent results (8–15). For example, a previous Korean study reported that a higher BMI was associated with an increased risk of deep or infratentorial CMBs, but not with lobar CMBs (8). In contrast, a more recent UK study found that a higher BMI was linked to a decreased risk of lobar CMBs, with no association observed for deep or infratentorial CMBs (9).

Most previous studies on the relationship between BMI and CMB have been cross-sectional (8, 9, 13–15), with only a limited number of longitudinal studies conducted (10–12). However, because cross-sectional comparisons capture only a single moment in time, they have limitations in establishing causal relationships. Moreover, among the few longitudinal studies, most focused solely on the total number of CMBs without considering their location and distribution, which may have contributed to the inconsistent results (11, 12). There is one longitudinal study that stratified CMBs by location and distribution, which found that baseline underweight was associated with an increased risk of lobar CMBs compared to normal weight, but not with deep (including infratentorial) CMBs (10).

Considering the sex differences in the effects of BMI on hemorrhagic stroke, we hypothesized that sex may also affect the relationship between BMI and CMB. Although previous studies have designated sex as a confounding factor (8–10), no study has focused on sex differences in the association between CMB and BMI. If sex is an important modifier, unrecognized sex differences may have contributed to the inconsistent findings of previous studies (8–10). Therefore, we aimed to conduct sex-stratified analyses to determine whether sex modifies the effect of BMI on CMB progression. In this study, we analyzed longitudinal data from the CHALLENGE study (Comparison Study of Cilostazol and Aspirin on Changes in Volume of Cerebral Small Vessel Disease White Matter Changes) (16) to investigate the association between BMI and CMB progression separately in men and women.

## Methods

### Study participants

This study was a sub-analysis of the CHALLENGE (Unique identifier: NCT01932203)^1^ trial, a multicenter, double-blind, randomized controlled trial that enrolled participants aged 50–85 years with cSVD (16). The diagnosis of cSVD was established based on the presence of at least one lacune and moderate to severe white matter hyperintensities (WMHs) according to the modified Fazekas criteria for periventricular WMHs with a cap or rim of ≥5 mm and deep WMHs with a maximum diameter of ≥10 mm (17). The main objective of the trial was to compare the effects of cilostazol and aspirin on the changes in WMHs volume over 2 years. Between July 2013 and August 2016, 282 participants were screened for eligibility, of whom 256 participants were randomly assigned to the cilostazol or aspirin group. Of the 256 CHALLENGE subjects, 189 participants with a follow-up magnetic resonance imaging (MRI) scan were included in our analysis. A comparison between subjects with and without a follow-up MRI scan is presented in Supplementary Table 1. There were no significant differences between the two groups, including age, sex, vascular risk factors, and baseline CMBs. This study was conducted in accordance with the guidelines of the Declaration of Helsinki and approved by the Institutional Review Board of Inha University Hospital (approval number: IUH-IRB 2013–03-006). Written informed consent was obtained from all potential participants prior to enrollment.

### Imaging markers

Brain MRI data including axial T2∗-weighted gradient-echo sequence (4 mm slice thickness with no interslice gap) were acquired using a 3.0 Tesla MR scanner. The same scanner and the same sequence were used for the baseline and follow-up MRI. CMBs were defined as lesions with a diameter of ≤10 mm (18) and rated using the Microbleed Anatomical Rating Scale (MARS) (19). Two experienced neurologists, blinded to the clinical information, counted CMBs in the lobar regions (frontal, parietal, temporal, occipital, and insular cortices) and in the deep/infratentorial regions (basal ganglia, thalamus, internal/external capsules, corpus callosum, deep/periventricular white matter, brainstem, and cerebellum) on gradient-echo MRI images for each patient. The Pearson’s correlation coefficient for the agreement on the number of CMBs between the two neurologists was 0.958 (95% confidence interval 0.809–0.989; *p* < 0.001). The two neurologists reached a consensus after discussion in cases of initial disagreement.

### Statistical analysis

Baseline characteristics were compared between men and women using the chi-square test for categorical variables (prevalence of hypertension, diabetes, dyslipidemia, and current smoking, BMI classification, proportion of apolipoprotein E4 (APOE4) carriers, types of antiplatelet medications, and proportion of individuals with CMBs). Student’s *t*-test was used for normally distributed continuous variables (age and BMI), while the Mann–Whitney U-test was applied for non-normally distributed continuous variables (follow-up duration, WMH volume, and counts of lacunes and CMBs) to compare men and women.

We used a generalized linear mixed model (GLMM) with a negative binomial distribution to evaluate the association between BMI and the longitudinal change in CMB count. The outcome variable, the 2-year change in CMB count, was count data exhibiting overdispersion (variance > mean). Therefore, a negative binomial GLMM was used instead of a Poisson model. Model 1 was adjusted for age and baseline CMB count, and Model 2 was further adjusted for hypertension, diabetes, dyslipidemia, current smoking, APOE4 status, and antiplatelet medication (aspirin vs. cilostazol). Sex-stratified analyses were performed to assess whether sex modifies the association between BMI and CMB progression.

All statistical analyses were performed using SPSS version 23.0 (IBM SPSS Inc., New York, NY, United States). Two-tailed *p* values were obtained, and *p* < 0.05 was considered statistically significant.

## Results

A total of 65 men and 124 women were included in the analysis. The baseline characteristics of men and women are presented in Table 1. Compared with men, women were older (74.5 vs. 71.5 years; *p* = 0.014). Men were more likely than women to be current smokers (15.4% vs. 1.6%; *p* < 0.001). No other baseline clinical differences, including BMI, were observed between the groups. A comparison of baseline cSVD markers revealed that the median [interquartile range (IQR)] number of lacunes was higher in men than in women [8 (4–13) vs. 5 (2–9); *p* = 0.014]. There were no significant differences in total WMHs volume, the prevalence of CMB at baseline (70.8% vs. 71.8%; *p* = 1.000), or the median median [IQR] number of total CMBs [1 (0–7) vs. 2 (0–7); *p* = 0.810].

**Table 1:** Comparison of baseline characteristics between men and women.

Characteristics	Men (*n* = 65)	Women (*n* = 124)	*P-*value
Age, years	71.5 (7.8)	74.5 (5.9)	0.014
Hypertension	52 (80.0%)	104 (83.9%)	0.548
Diabetes	27 (41.5%)	46 (37.1%)	0.637
Dyslipidemia	33 (50.8%)	60 (48.4%)	0.759
Current smoking	10 (15.4%)	2 (1.6%)	<0.001
Body mass index, kg/m^2^	24.2 (2.7)	25.0 (3.2)	0.085
BMI classification			0.194
Underweight (<18.5 kg/m^2^)	2 (3.1%)	0 (0.0%)	
Normal weight (18.5–22.9 kg/m^2^)	17 (26.2%)	30 (24.2%)	
Overwieght (23–24.9 kg/m^2^)	20 (30.8%)	34 (27.4%)	
Obese (≥25 kg/m^2^)	26 (40.0%)	60 (48.4%)	
Apolipoprotein E4 carrier	19 (29.2%)	30 (24.2%)	0.602
Antiplatelet medication			0.542
Aspirin	33 (50.8%)	69 (55.6%)	
Cilostazol	32 (49.2%)	55 (44.4%)	
Follow-up, years	1.99 (1.98–2.02)	2.00 (1.98–2.02)	0.998
Baseline cSVD markers			
WMHs volume, mL	34.5 (20.0–47.7)	34.8 (26.5–50.0)	0.262
Number of lacunes	8 (4–13)	5 (2–9)	0.014
Presence of CMBs	46 (70.8%)	89 (71.8%)	1.000
Strictly lobar	11 (16.9%)	16 (12.9%)	0.150
Strictly deep	10 (15.4%)	24 (19.4%)	0.257
Mixed	25 (38.5%)	49 (39.5%)	1.000
Number of CMBs			
Deep/infratentorial	1 (0–4)	1 (0–5)	0.291
Lobar	1 (0–3)	0 (0–2)	0.407
Total	1 (0–7)	2 (0–7)	0.810

Values are presented as the percentage (%), mean (SD), or median (IQR). CMB, cerebral microbleed; cSVD, cerebral small vessel disease; IQR, interquartile range; SD, standard deviation; WMH, white matter hyperintensity. *p < 0.05.

**Figure 1:**
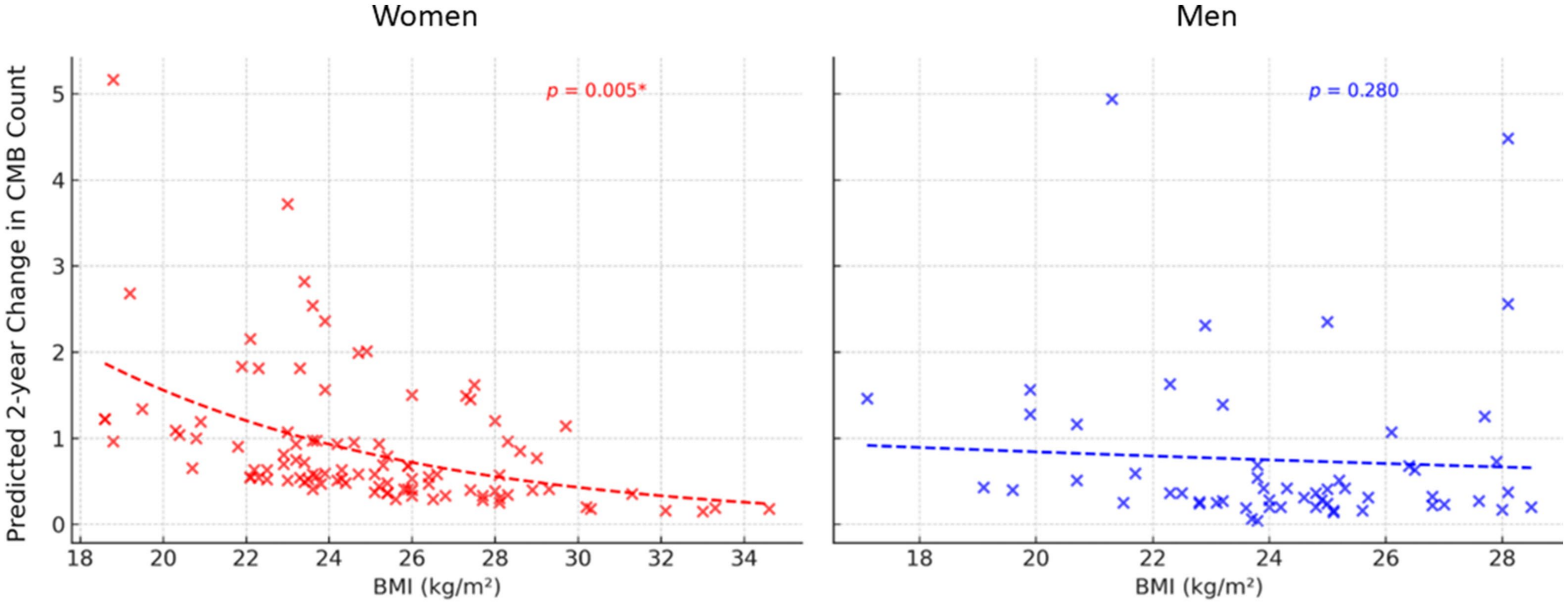
Predicted 2-year change in cerebral microbleed (CMB) count by body mass index (BMI) in women and men. Results were obtained using a generalized linear mixed model with a negative binomial distribution, adjusted for age and baseline CMB count. *p < 0.05.

In the sex-stratified negative binomial mixed-effects model, a significant association between BMI and 2-year changes in CMB counts was observed only in women, but not in men (Table 2). Among women, each 1 kg/m^2^ increase in BMI was significantly associated with a decrease in the 2-year change in the total number of CMBs [Model 1: *β* = −0.120, 95% confidence interval (CI): −0.202 to −0.037, *p* = 0.005; Model 2: *β* = −0.117, 95% CI: −0.203 to −0.032, *p* = 0.007]. When CMBs were categorized as lobar or deep/infratentorial, significant associations were observed for both lobar CMBs (Model 1: *β* = −0.114, 95% CI: −0.213 to −0.015, *p* = 0.024; Model 2: β = −0.112, 95% CI: −0.216 to −0.009, *p* = 0.034) and deep/infratentorial CMBs (Model 1: β = −0.123, 95% CI: −0.222 to −0.023, *p* = 0.015; Model 2: β = −0.123, 95% CI: −0.227 to −0.019, *p* = 0.020). In men, however, no significant association was observed between BMI and the 2-year change in CMB counts (all *p* > 0.05). Figure 1 shows the estimated effects of BMI on 2-year longitudinal changes in the total number of CMBs in women and men.

**Table 2:** Sex-specific association between body mass index (BMI) and 2-year change in cerebral microbleed (CMB) count.

2-year change in CMB count per 1-kg/m^2^ BMI increase						
	Total CMBs		Lobar CMBs		Deep/infratentorial CMBs	
	β (95% CI)	*P-*value	β (95% CI)	*P-*value	β (95% CI)	*P-*value
Model 1^†^						
Women	−0.120 (−0.202, −0.037)	0.005^*^	−0.114 (−0.213, −0.015)	0.024^*^	−0.123 (−0.222, −0.023)	0.015^*^
Men	−0.072 (−0.202, 0.058)	0.280	−0.111 (−0.264, 0.042)	0.156	−0.003 (−0.188, 0.181)	0.973
Model 2^‡^						
Women	−0.117 (−0.203, −0.032)	0.007^*^	−0.112 (−0.216, −0.009)	0.034^*^	−0.123 (−0.227, −0.019)	0.020^*^
Men	−0.000021 (−0.164, 0.164)	1.000	−0.032 (−0.228, 0.165)	0.752	0.036 (−0.185, 0.256)	0.750

CMBs, cerebral microbleeds; CI, confidence interval; BMI, body mass index. ^*^p < 0.05. ^†^Results of a generalized linear model (negative binomial) adjusted for the baseline number of CMBs and age. ^‡^Results of a generalized linear model (negative binomial) adjusted for the baseline number of CMBs, age, hypertension, diabetes, dyslipidemia, current smoking, apolipoprotein E4, and antiplatelet medication (cilostazol vs. aspirin).

To minimize potential ceiling effects, we conducted a sensitivity analysis excluding participants with >10 baseline CMBs. The results were consistent with the main analysis, showing similar trends without meaningful differences (Supplementary Table 2). Additionally, a sensitivity analysis excluding the small number of midlife participants (<65 years; 9 men and 6 women) did not change the observed trends or affect statistical significance (Supplementary Table 3).

## Discussion

In this study, we examined the impact of BMI on CMB progression in men and women separately, using longitudinal data from the CHALLENGE study, which included patients aged 50–85 years with cSVD. Two key considerations should be noted when interpreting our findings. First, the CHALLENGE trial, which served as the basis for our analysis, was not designed to examine sex-specific differences, and the smaller number of men may have limited the statistical power for male-specific analyses. Second, only baseline BMI was available because repeated measurements during the 2-year follow-up were not collected. Therefore, our findings should be interpreted as reflecting the association between baseline BMI and subsequent CMB progression, without accounting for potential changes in BMI over time.

Our analysis showed that BMI was associated with CMB progression only in women. Among women, higher BMI was associated with a lower rate of CMB progression. In previous studies evaluating the association between BMI and CMB (8–10), sex was treated as a confounder, but stratified analyses by sex were not performed. Our study was designed under the assumption that sex differences might exist; therefore, sex-stratified analyses were planned. Notably, in our study population, when adjusting for sex without stratification, there seemed to be a significant association between BMI and CMB progression in both men and women. If our primary goal had not been to investigate sex differences, these differences within our population might have remained undetected. Our findings suggest that sex could be a potential modifier in the association between BMI and CMB.

Our study found that higher BMI was associated with less CMB progression in women, which may be in line with findings from a previous UK study reporting an association between higher BMI and a lower risk of hemorrhagic stroke (3). The mechanism underlying this inverse relationship between BMI and CMB progression remains unclear; however, one possible explanation could involve estrogen. Most women in our study, who were 56–85 years of age (mean 74.5 years), were likely postmenopausal, and postmenopausal estrogen levels have been reported to be higher in obese women than in those with normal weight (20, 21). Estrogen is known to exert a protective effect on vascular endothelial cells by reducing oxidative stress (22, 23). Estrogen-mediated vascular protection may partly contribute to the reduced risk of CMB progression in women with higher BMI in later life. Nevertheless, the lack of precise data on menopausal status, information on the use of hormonal therapy, and actual estrogen levels poses a limitation in supporting this proposed mechanism. Additionally, findings in younger age groups may differ from ours. In late-life, higher BMI in women may be linked to relatively higher estrogen levels, which could contribute to vascular protection. In contrast, midlife obesity is associated with adverse metabolic and vascular effects, such as hypertension, dyslipidemia, and insulin resistance, potentially increasing the risk of cerebrovascular disease (24). Therefore, further age-specific studies are warranted.

Unlike in women, the lack of a significant association between BMI and CMB progression in men remains unclear. As noted above, the limited statistical power of the male subgroup in our study should be taken into consideration. Beyond this, sex-specific biological factors may also contribute. Because our study enrolled relatively older adults, the observed sex-specific associations may reflect later life vascular aging patterns, with accelerated vascular changes in women after menopause and earlier aging in men (25, 26). In this context, higher BMI in late life may confer vascular protection in women, possibly through mechanisms such as preserved estrogen levels, whereas such effects appear to be less evident in men. Sex differences in adipokine profiles could also partly contribute to the sex-specific findings in our study. Even at the same BMI, men tend to exhibit a less favorable adipokine profile than women, characterized by lower adiponectin levels and a more pro-inflammatory state (27). Moreover, previous studies have suggested that for the same BMI increase, the decline in adiponectin may be greater in men than in women (28). However, further research is warranted to clarify the association between BMI and CMB progression in men because of the limited statistical power of the male subgroup in our study.

In the analysis stratified by CMB location, significant associations were observed for both deep and lobar CMBs in women. Deep CMBs are generally attributed to cerebrovascular risk factors such as hypertensive angiopathy (6). In contrast, lobar CMBs have heterogeneous underlying pathologies: strictly lobar CMBs are most commonly associated with CAA (6), whereas mixed-type lobar CMBs are more frequently related to hypertensive angiopathy (29, 30). Moreover, some mixed-type lobar CMBs may reflect an overlap between CAA and hypertensive angiopathy (31). We did not have data on amyloid positivity, and this uncertainty regarding the underlying pathology of lobar CMBs is one of the limitations of our study. Future studies using amyloid PET or CSF amyloid are warranted to investigate the association between BMI and lobar CMBs based on their underlying pathology.

This was the first study to focus on sex differences in the relationship between BMI and CMB. A key strength of our study was its longitudinal design rather than a cross-sectional design. However, several limitations should be acknowledged. First, the overall sample size of this study was relatively small. As mentioned earlier, the smaller number of men compared to women may have limited the statistical power of the male-specific analyses. Indeed, based on the Wald chi-square statistic, the statistical power appeared adequate in women but limited in men. Therefore, future studies with larger sample sizes, balanced sex ratios, and longer follow-up periods are warranted to confirm and extend these findings. Second, only baseline BMI data were available, and changes in BMI during the 2-year follow-up were not assessed. Sex-related baseline differences in age and smoking status may also pose potential limitations. Finally, because all participants were Korean, the generalizability of our findings to non-Asian populations may be limited.

## Conclusion

Higher BMI in later life was associated with less CMB progression in women, but not in men. These findings highlight sex as a potential modifier in the relationship between BMI and CMB progression, underscoring the need for larger, biomarker-based longitudinal studies to confirm these results and clarify the underlying mechanisms.

## Data Availability

The raw data supporting the conclusions of this article will be made available by the authors, without undue reservation.

## Footnotes

^1^ Clinicaltrials.gov
