# Supplementary material for: Sex-specific association between body mass index and cerebral microbleed progression in adults aged 50–85 years
Source: Front Neurol. 2025 Sep 8;16:1624905. doi: 10.3389/fneur.2025.1624905 (PMC12450651; doi:10.3389/fneur.2025.1624905)
Supplement: Supplementary file 1 [file Table_1.docx]

**Supplementary Table 1. Comparison between subjects with and without a follow-up magnetic resonance imaging scan**

|  | With a follow-up scan (*n* = 189) | Without a follow-up scan (*n* = 67) | *P* value |
| --- | --- | --- | --- |
| Age, years | 73.4 (6.8) | 74.8 (6.2) | 0.173 |
| Sex, female | 124 (65.6%) | 39 (58.2%) | 0.303 |
| Hypertension | 156 (82.5%) | 53 (79.1%) | 0.582 |
| Diabetes | 72 (38.1%) | 34 (50.7%) | 0.071 |
| Dyslipidemia | 93 (49.2%) | 33 (49.3%) | 1.000 |
| Current Smoking | 12 (6.3%) | 7 (10.4%) | 0.284 |
| Body mass index, kg/m^2^ | 24.7 (3.1) | 24.5 (3.5) | 0.505 |
| CMBs | *n* = 189 | *n* = 65 |  |
| Presence of CMBs | 135 (71.4%) | 40 (61.5%) | 0.162 |
| Number of CMBs |  |  |  |
| Deep/infratentorial | 1 (0-4) | 1 (0-3) | 0.606 |
| Lobar | 1 (0-2) | 0 (0-3) | 0.678 |
| Total | 2 (0-7) | 2 (0-6) | 0.559 |

The values are presented as number (%), mean (SD), or median (IQR). CMB, cerebral microbleed; SD, standard deviation; IQR, interquartile range.

**Supplementary Table 2. Sex-specific association between body mass index (BMI) and 2-year change in cerebral microbleed (CMB) count: results from a sensitivity analysis excluding participants with >10 total CMBs**

| **2-year change in CMB count per 1-kg/m² BMI increase** | | | | | | |
| --- | --- | --- | --- | --- | --- | --- |
|  | ***Total CMBs*** | | ***Lobar CMBs*** | | ***Deep/infratentorial CMBs*** | |
|  | β (95% CI) | *P* value | β (95% CI) | *P* value | β (95% CI) | *P* value |
| ***Model 1^†^*** | | | | | | |
| **Women (n=101)** | -0.151  (-0.257, -0.044) | 0.005^*^ | -0.152  (-0.284, -0.020) | 0.024^*^ | -0.144  (-0.268, -0.020) | 0.023^*^ |
| **Men (n=57)** | 0.028  (-0.134, 0.191) | 0.731 | -0.020  (-0.202, 0.163) | 0.834 | 0.132  (-0.122, 0.387) | 0.308 |
| ***Model 2^‡^*** | | | | | | |
| **Women (n=101)** | -0.168  (-0.277, -0.059) | 0.002^*^ | -0.174  (-0.313, -0.034) | 0.015^*^ | -0.168  (-0.299, -0.036) | 0.012^*^ |
| **Men (n=57)** | 0.084  (-0.110, 0.278) | 1.000 | -0.020  (-0.241, 0.201) | 0.860 | 0.188  (-0.109, 0.485) | 0.215 |

CMBs, cerebral microbleeds; CI, confidence interval; BMI, body mass index.

^*^*P* < 0.05

^†^Results of a generalized linear model (negative binomial) adjusted for the baseline number of CMBs and age.

^‡^Results of a generalized linear model (negative binomial) adjusted for the baseline number of CMBs, age, hypertension, diabetes, dyslipidemia, current smoking, apolipoprotein E4, and antiplatelet medication (cilostazol vs. aspirin).

**Supplementary Table 3. Sex-specific association between body mass index (BMI) and 2-year change in cerebral microbleed (CMB) count: results from a sensitivity analysis excluding participants younger than 65 years**

| **2-year change in CMB count per 1-kg/m² BMI increase** | | | | | | |
| --- | --- | --- | --- | --- | --- | --- |
|  | ***Total CMBs*** | | ***Lobar CMBs*** | | ***Deep/infratentorial CMBs*** | |
|  | β (95% CI) | *P* value | β (95% CI) | *P* value | β (95% CI) | *P* value |
| ***Model 1^†^*** | | | | | | |
| **Women (n=118)** | -0.197  (-0.325, -0.068) | 0.003^*^ | -0.105  (-0.187, -0.023) | 0.012^*^ | -0.089  (-0.153, -0.025) | 0.007^*^ |
| **Men (n=56)** | -0.060  (-0.236, 0.116) | 0.503 | -0.069  (-0.217, 0.079) | 0.361 | 0.007  (-0.049, 0.063) | 0.804 |
| ***Model 2^‡^*** | | | | | | |
| **Women (n=118)** | -0.190  (-0.322, -0.057) | 0.005^*^ | -0.097  (-0.180, -0.013) | 0.024^*^ | -0.090  (-0.157, -0.024) | 0.008^*^ |
| **Men (n=56)** | -0.030  (-0.219, 0.159) | 0.759 | -0.014  (-0.159, 0.132) | 0.855 | 0.007  (-0.053, 0.068) | 0.814 |

CMBs, cerebral microbleeds; CI, confidence interval; BMI, body mass index.

^*^*P* < 0.05

^†^Results of a generalized linear model (negative binomial) adjusted for the baseline number of CMBs and age.

^‡^Results of a generalized linear model (negative binomial) adjusted for the baseline number of CMBs, age, hypertension, diabetes, dyslipidemia, current smoking, apolipoprotein E4, and antiplatelet medication (cilostazol vs. aspirin).
